# Low-Dose Piperlongumine Rescues Impaired Function of Endothelial Progenitor Cells and Reduces Cerebral Ischemic Injury in High-Fat Diet-Fed Mice

**DOI:** 10.3389/fphar.2021.689880

**Published:** 2021-11-15

**Authors:** Xiao-Hui Dong, Cheng Peng, Yu-Yi Zhang, Yu Jiang, Li-Jun Yang, Jia-Bei He, Xia Tao, Chuan Zhang, Alex F Chen, He-Hui Xie

**Affiliations:** ^1^ Institute for Developmental and Regenerative Cardiovascular Medicine, Xinhua Hospital, Shanghai Jiao Tong University School of Medicine, Shanghai, China; ^2^ Department of Pharmacy, Shanghai East Hospital, Tongji University School of Medicine, Shanghai, China; ^3^ School of Public Health and Hongqiao International Institute of Medicine, Shanghai Jiao Tong University School of Medicine, Shanghai, China; ^4^ Department of Pharmacy, Shanghai Changzheng Hospital, Second Military Medical University, Shanghai, China; ^5^ School of Medicine, Shanghai University, Shanghai, China

**Keywords:** piperlongumine, endothelial progenitor cells, angiogenesis, cerebral ischemic injury, hyperlipidemia

## Abstract

It is of great clinical significance to develop potential novel strategies to prevent cardio-cerebrovascular complications in patients with hyperlipidemia. Vascular Endothelial integrity and function play a key role in the prevention of cardio-cerebrovascular diseases. Endothelial progenitor cells (EPCs) can home to sites of ischemic injury and promote endothelial regeneration and neovascularization. Hypercholesterolemia impairs the function of EPC. The present study attempted to identify the effect of piperlongumine on EPCs’ angiogenic potential and cerebral ischemic injury in high-fat diet-fed (HFD-fed) mice. Here, we showed that treatment with low-does piperlongumine (0.25 mg/kg/day) for 8 weeks significantly improved EPCs function and reduced the cerebral ischemic injury (both infarct volumes and neurobehavioral outcomes) in HFD-fed mice. In addition, low-dose piperlongumine administration increased intracellular NO level and reduced intracellular O_2_^-^ level in EPCs of HFD-fed mice. Moreover, incubation with piperlongumine (1.0 μM, 24 h) reduced thrombospondin-1/2 (TSP-1/2, a potent angiogenesis inhibitor) expression levels in EPCs from HFD-fed mice, increased the therapeutic effect of EPC from HFD-fed mice on cerebral ischemic injury reduction and angiogenesis promotion in HFD-fed mice, and the donor derived EPCs homed to the recipient ischemic brain. In conclusion, low-dose piperlongumine can enhance EPCs’ angiogenic potential and protect against cerebral ischemic injury in HFD-fed mice. It is implied that treatment with low-dose piperlongumine might be a potential option to prevent ischemic diseases (including stroke) in patients with hyperlipidemia, and priming with piperlongumine might be a feasible way to improve the efficacy of EPC-based therapy for ischemic diseases.

## Introduction

Dyslipidemia is an important risk factor for ischemic cerebrovascular disease (Kopin and Lowenstein, 2017). Nowadays, statins have been the first choice lipid-modifying medications for preventing and treating cardio-cerebrovascular diseases (Amarenco et al., 2004; Baigent et al., 2005). However, despite the widespread clinical use of statin therapy, patients with dyslipidaemia suffer substantial morbidity and mortality from cardio-cerebrovascular disease (Amarenco et al., 2006; Chapman et al., 2010). Accordingly, it is of great clinical importance to develop new strategies to prevent cardio-cerebrovascular complications in patients with hyperlipidemia.

Vascular Endothelial integrity and function play a key role in the prevention of cerebrovascular diseases, such as stroke (a leading cause of disability and death in the world) (Versari et al., 2009; Zhao et al., 2013). Endothelial progenitor cells (EPCs) are a population of new cells released into peripheral blood by bone marrow to promote endothelial repair and neovasculogenesis in response to an ischaemic injury (Bayraktutan, 2019). Accumulating evidence indicate that EPCs may be of pivotal importance to help determine the extent of functional recovery. EPC-based cell therapy is now considered as an important novel therapeutic treatment for stroke (Fan et al., 2010; Zhao et al., 2013; Dong et al., 2017; Peng et al., 2018). Thus, it can be logically speculated that promoting EPC function and EPC-mediated ischemic angiogenesis may serve as a promising strategy to prevent cardio-cerebrovascular complications (such as coronary heart disease, stroke, and so on) in patients with hyperlipidemia.

Long pepper, the grown ear-fruit, is widely used in traditional Mongolian medicine for treating coronary heart disease and hyperlipidemia (Bao et al., 2012). Piperlongumine is a primary constituent isolated from the plant species long pepper. Studies have suggested that piperlongumine has various biological functions such as anti-platelet aggregation, anti-inflammatory, and anti-cancer properties (Park et al., 2008; Golovine et al., 2013; Kim et al., 2018). The barrier protective activities of piperlongumine were determined in LPS-activated human umbilical vein endothelial cells (HUVECs) and in mice (Lee et al., 2013). However, piperlongumine’s function in preventing cardio-cerebrovascular complications in patients with hyperlipidemia has not been studied.

Based on these findings, this study sought to test the hypothesis that piperlongumine could reduce cerebral ischemic injury by promoting EPC-mediated angiogenesis in HFD-fed mice.

Study showed that oral administration of GB-N (a new N-isobutylamine product of piperine derivative) at doses of 2.5–10 mg/kg/day for 14 days could significantly reduce the level of serum triacylglycerols and elevate the level of serum high-density lipoprotein cholesterol in hyperlipidemic rats (Bao et al., 2012). In the current study, we found that low-dose piperlongumine (0.25 mg/kg/day) did not affect blood lipid levels. The present study, however, demonstrated for the first time that intraperitoneal low-dose piperlongumine injection for eight consecutive weeks significantly reduced cerebral ischemic injury in HFD-fed mice, which might be partly due to the promotion of EPC-mediated angiogenesis.

## Methods

### Animals and Treatment

**FIGURE 1:**
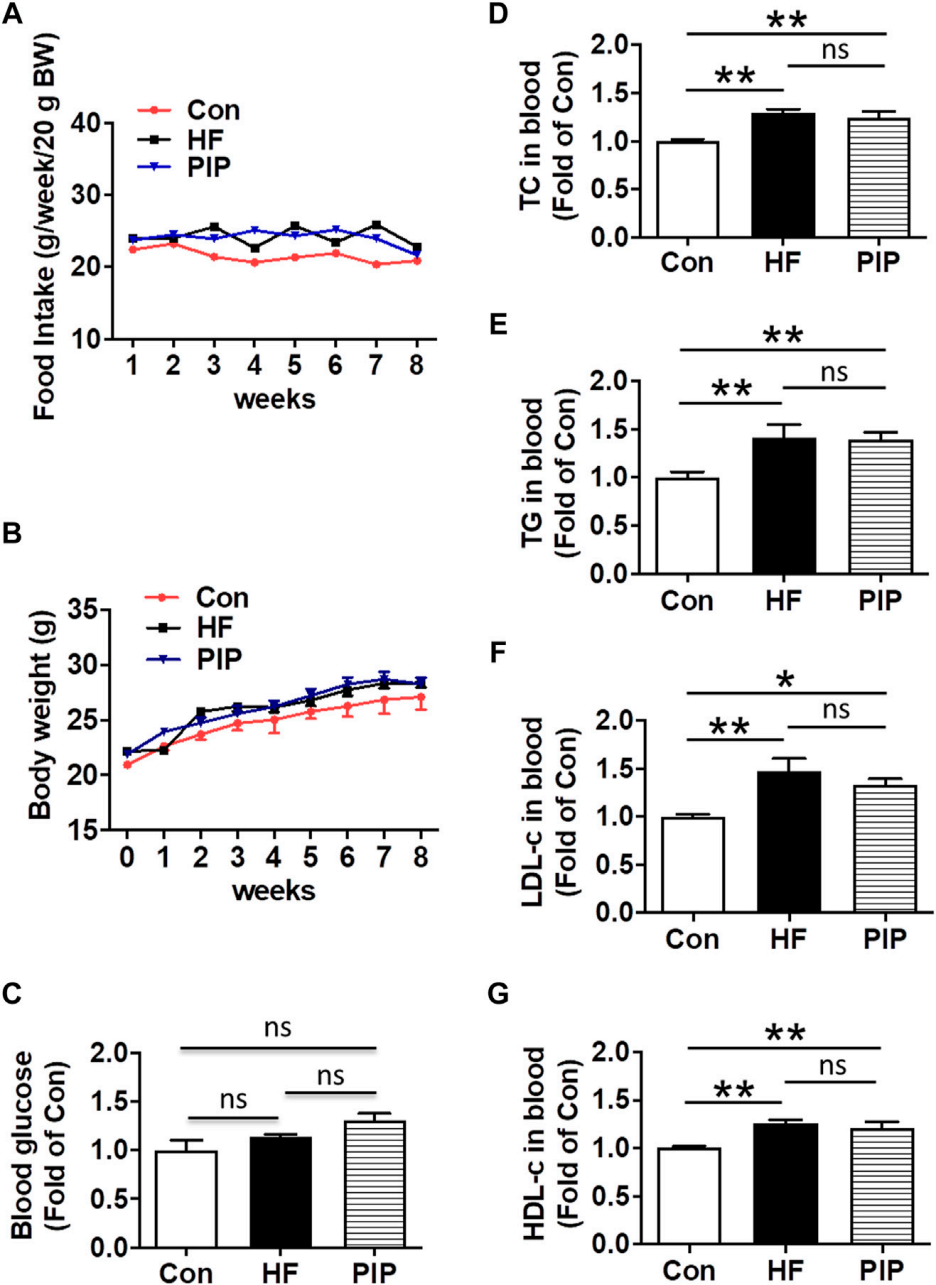
Effects of low-dose piperlongumine administration on food intake, body weight, fasting blood glucose and lipid levels in HFD-fed mice. Male C57BL/6J mice at 8 weeks of age were randomly allocated to three groups and fed with high-fat rodent chow with or without receiving an intraperitoneal injection of low-does piperlongumine (0.25 mg/kg/d) for 8 weeks. After 8 weeks treatment, the mice were subjected to various analyses. **(A)**, Food intake during the 8 weeks experiments (n = 8–11). **(B)**, Body weight during the 8 weeks experiments (n = 7–11). **(C)**, Effect of piperlongumine on fasting blood glucose (n = 7–9). **(D–F)**, Blood total cholesterol (TC) levels, triglyceride (TG) levels, low-density lipoprotein cholesterol (LDL-c) levels, and high-density lipoprotein cholesterol (HDL-c) levels after 8 weeks treatment (n = 5–9). Throughout, bars represent means; error bars represent SEM. Values were normalized to Con. *p < 0.05, **p < 0.01. Con, control mice; HF, HFD-fed mice; PIP, HFD-fed mice treated with piperlongumine.

Male C57BL/6 mice (10–12 weeks, 20–25 g) were obtained from Shanghai SLAC Laboratory Animal Co., Ltd (Shanghai, China). Mice were maintained in the following conditions: temperature: 23–25 °C, relative humidity: 45–65%, and light cycle: 12 h light/12 h dark cycle. Previous studies reported that estrogens exert neuroprotective effects in an animal model of ischemia (Simpkins et al., 1997; Suzuki et al., 2009). To avoid the interference of estrogen on ischemic stroke, only male mice were selected in this study. Piperlongmine was purchased from Indofine Chemical Company (Hillsborough, United States). The high-fat rodent chow diet contained 20.4% lard, 15% sucrose, 12.3% casein, 2% premix, 0.8% maltodextrin and 49.5% standard diet was provided by Shanghai Pu Lu Teng Biological Technology Co., Ltd., China. The mice were allowed 7 days for habituation and were fed with standard rodent chow. At the beginning of the experiment, male mice were randomly allocated to three groups and allowed free access to water and different types of diets. The mice on standard rodent chow diet were injected intraperitoneally daily with vehicle (saline solution; Con) for eight consecutive weeks. Meanwhile, the mice on high-fat rodent chow diet were injected intraperitoneally daily with vehicle (saline solution; HF), or low-dose piperlongmine (0.25 mg/kg/day; PIP) for eight consecutive weeks. The food intake and the body weight were measured from the beginning of the high-fat diet treatment and monitored weekly thereafter. The levels of fasting blood glucose, the serum levels of total cholesterol (TC), triacylglycerols (TG), low-density lipoprotein cholesterol (LDL-c) and high-density lipoprotein cholesterol (HDL-c) were measured after 8 weeks of treatment (Figure 1) (Tang et al., 2011).

**FIGURE 2:**
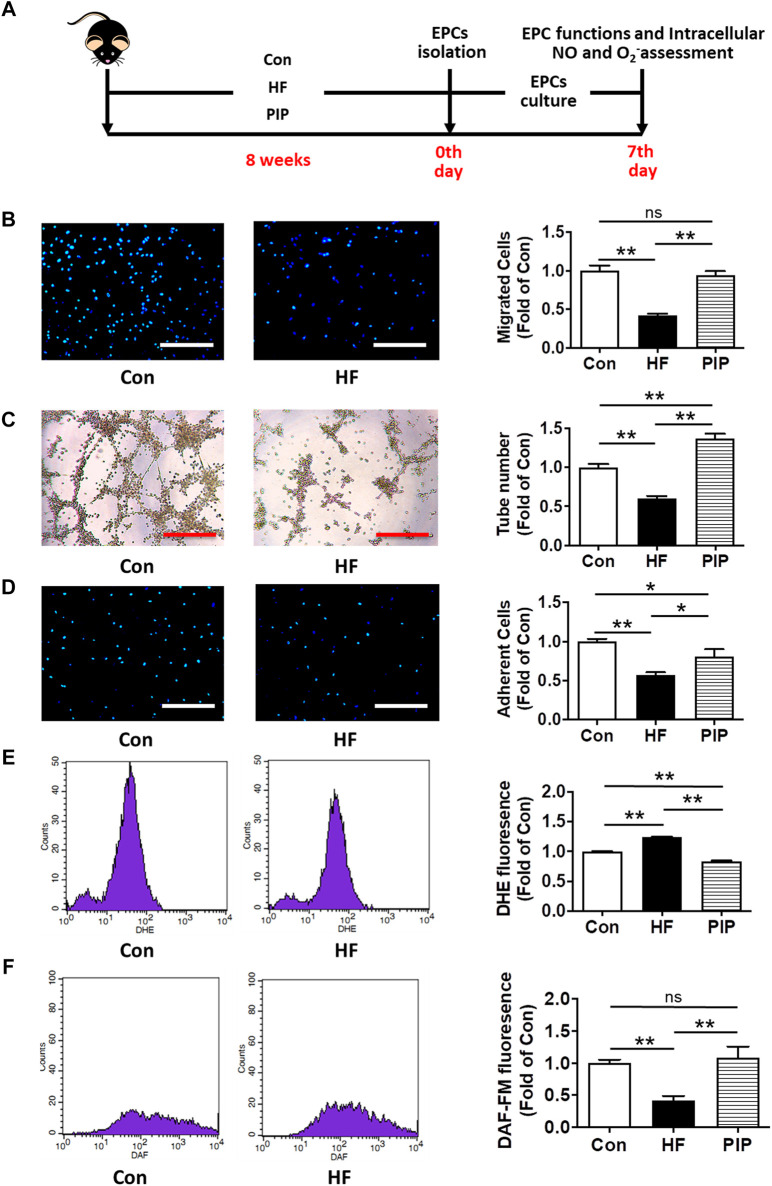
Low-dose Piperlongumine administration rescued EPC dysfunctions in HFD-fed mice. **(A)**, Experimental protocols: Male C57BL/6 mice (8 weeks) were randomized into three groups and fed with high-fat rodent chow with or without receiving an intraperitoneal injection of low-does piperlongumine (0.25 mg/kg/d) for 8 weeks. Then, the EPCs were isolated, cultured and examined in HFD-fed mice. **(B)**, EPC migration assay (n = 15–16). **(C)**, EPC tube formation assay (n = 20 per group). **(D)**, EPC adhesion assay (n = 20–26). **(E)**, Intracellular superoxide level of EPCs assessed by DHE staining flow cytometry (n = 4–5). **(F)**, Intracellular NO level of EPCs assessed by DAF-FM staining flow cytometry (n = 4–5). Throughout, bars represent means; error bars represent SEM. Values were normalized to Con. *p < 0.05, **p < 0.01. Con, control mice; HF, HFD-fed mice; PIP, HFD-fed mice treated with piperlongumine. Scale bar: 200 μm.

**FIGURE 3:**
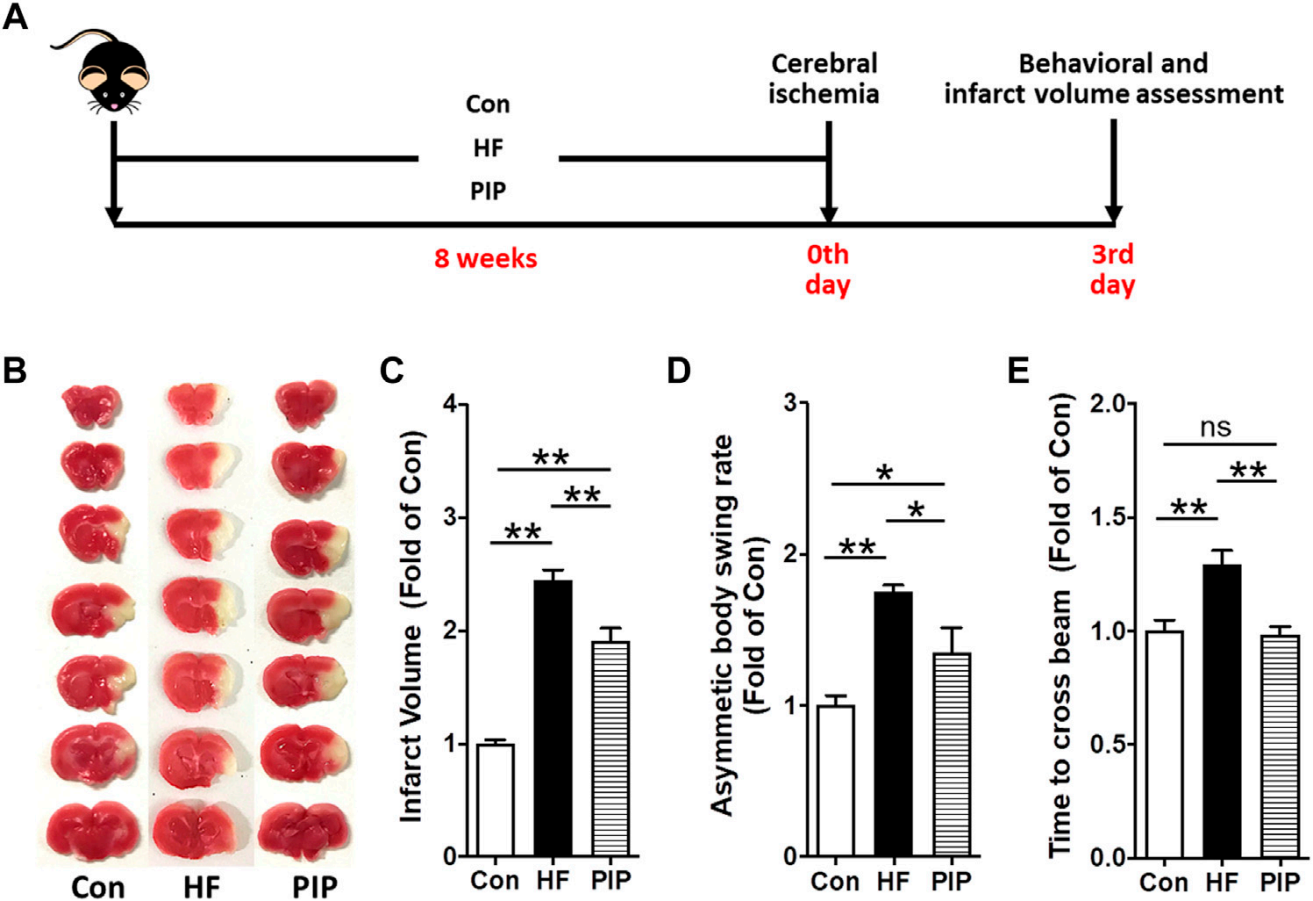
Low-dose Piperlongumine administration protected against cerebral ischemic injury in HFD-fed mice. **(A)**, Surgical protocols: Male C57BL/6 mice (8 weeks) were randomized into three groups and fed with high-fat rodent chow with or without receiving an intraperitoneal injection of low-does piperlongumine (0.25 mg/kg/d) for 8 weeks. Then the mice were subjected to focal cerebral ischemia by permanent. On day 3 after cerebral ischemia, behavioral test (including Body Asymmetry Test and Beam Test) was performed, and then the cerebral infarct volumes were determined. **(B,C)**, Images are representative of TTC-stained brain sections **(B)** and cerebral infarct volumes **(C)**. **(D,E)**, Neurobehavioral outcomes: Body Asymmetry Test **(D)** and Beam Test **(E)**. Throughout, bars represent means; error bars represent SEM. Values were normalized to Con. *p < 0.05, **p < 0.01. n = 8 per group. Con, control mice; HF, HFD-fed mice; PIP, HFD-fed mice treated with piperlongumine.

**FIGURE 4:**
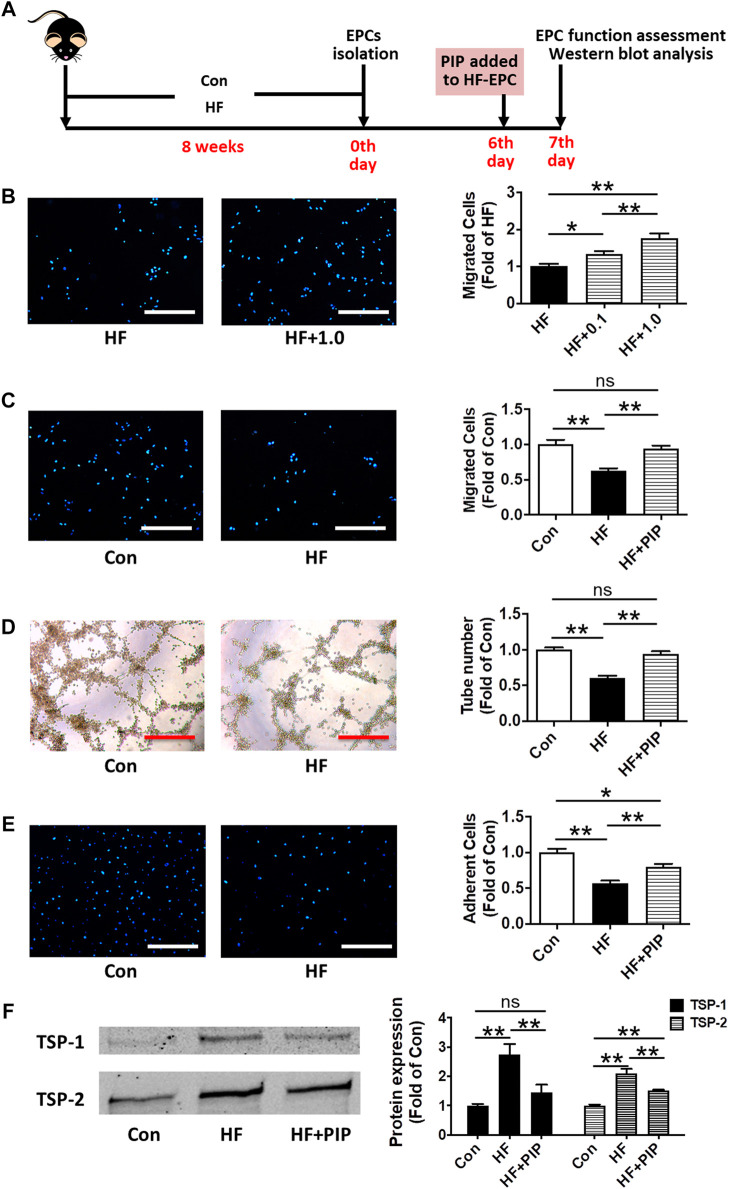
Incubation with piperlongumine rescued the impaired functions of EPCs from HFD-fed mice. **(A)**, Surgical protocols: Male C57BL/6 mice (8 weeks) were fed with high-fat rodent chow for 8 weeks. Then, the EPCs were isolated from the HFD-fed mice. After 6 days, piperlongumine was added to EPCs. Then, EPC functions were measurement at the 7^th^ day. **(B)**, Effects of piperlongumine (0.1 and 1.0µM, 24 h) on migration function of EPCs from HFD-fed mice (n = 15 per group). **(C)**, EPC migration assay (n = 13 per group). **(D)**, Tube formation assay of EPCs (n = 15–20). **(E)**, Adhesion assay of EPCs (n = 17–20). **(F)**, the representative images and the protein expression levels of TSP-1 (n = 7) and TSP-2 (n = 6). Throughout, bars represent means; error bars represent SEM. Values were normalized to Con or HF. *p < 0.05, **p < 0.01. Con, control EPC; HF, EPC from HFD-fed mice; HF+0.1, piperlongumine (0.1µM, 24 h)-incubated EPC from HFD-fed mice; HF+1.0, piperlongumine (1.0µM, 24 h)-incubated EPC from HFD-fed mice . HF + PIP, piperlongumine (1.0µM, 24 h)-incubated EPC from HFD-fed mice. Scale bar: 200 μm.

**FIGURE 5:**
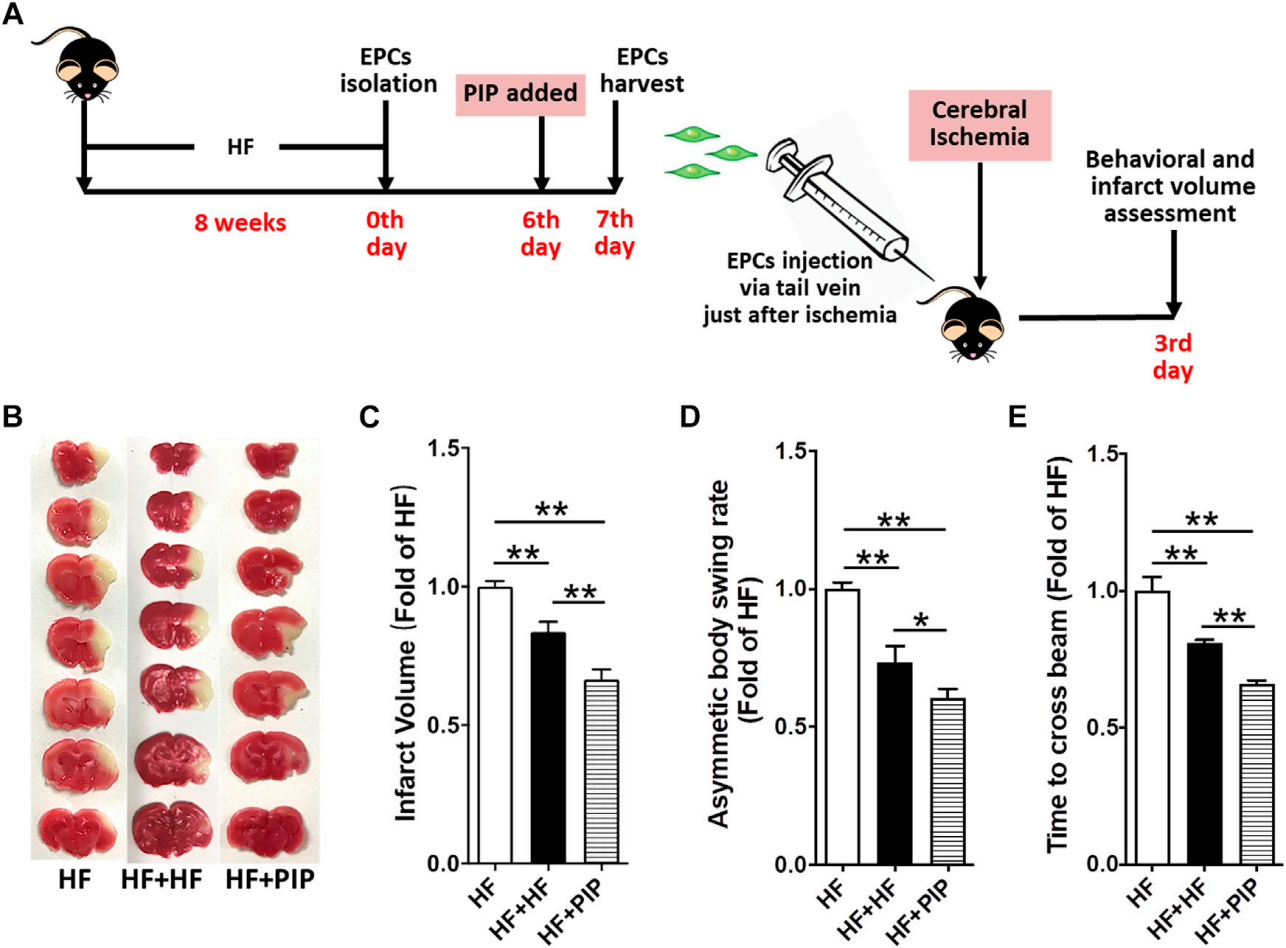
Incubation with piperlongumine increased the therapeutic effect of EPC from HFD-fed mice on cerebral ischemic injury reduction in HFD-fed mice. **(A)**, Surgical protocols: Male C57BL/6 mice (8 weeks) were fed with high-fat rodent chow for 8 weeks. Then, the EPCs were isolated from the HFD-fed mice. After 6 days, piperlongumine (1.0 μM) was added to EPCs for 24 h. EPCs were harvested at the 7^th^ day and injected into the HFD-fed mice *via* tail vein just after the cerebral ischemia (1×10^6^ EPCs per mice). Behavioral test and the infarct volumes were assessed on day 3 after middle cerebral artery occlusion. **(B,C)**, Images are representative of 2,3,5-triphenyltetrazolium chloride-stained brain sections **(B)** and cerebral infarct volumes **(C)**. **(D,E)**, Neurobehavioral outcomes: Body Asymmetry Test **(D)** and Beam Test **(E)**. Throughout, bars represent means; error bars represent SEM. Values were normalized to HF. *p < 0.05, **p < 0.01. n = 8-9 per group. HF, HFD-fed mice; HF + HF, HFD-fed mice treated with EPCs; HF + PIP, HFD-fed mice treated with piperlongumine (1.0µM, 24 h)-incubated EPCs.

**FIGURE 6:**
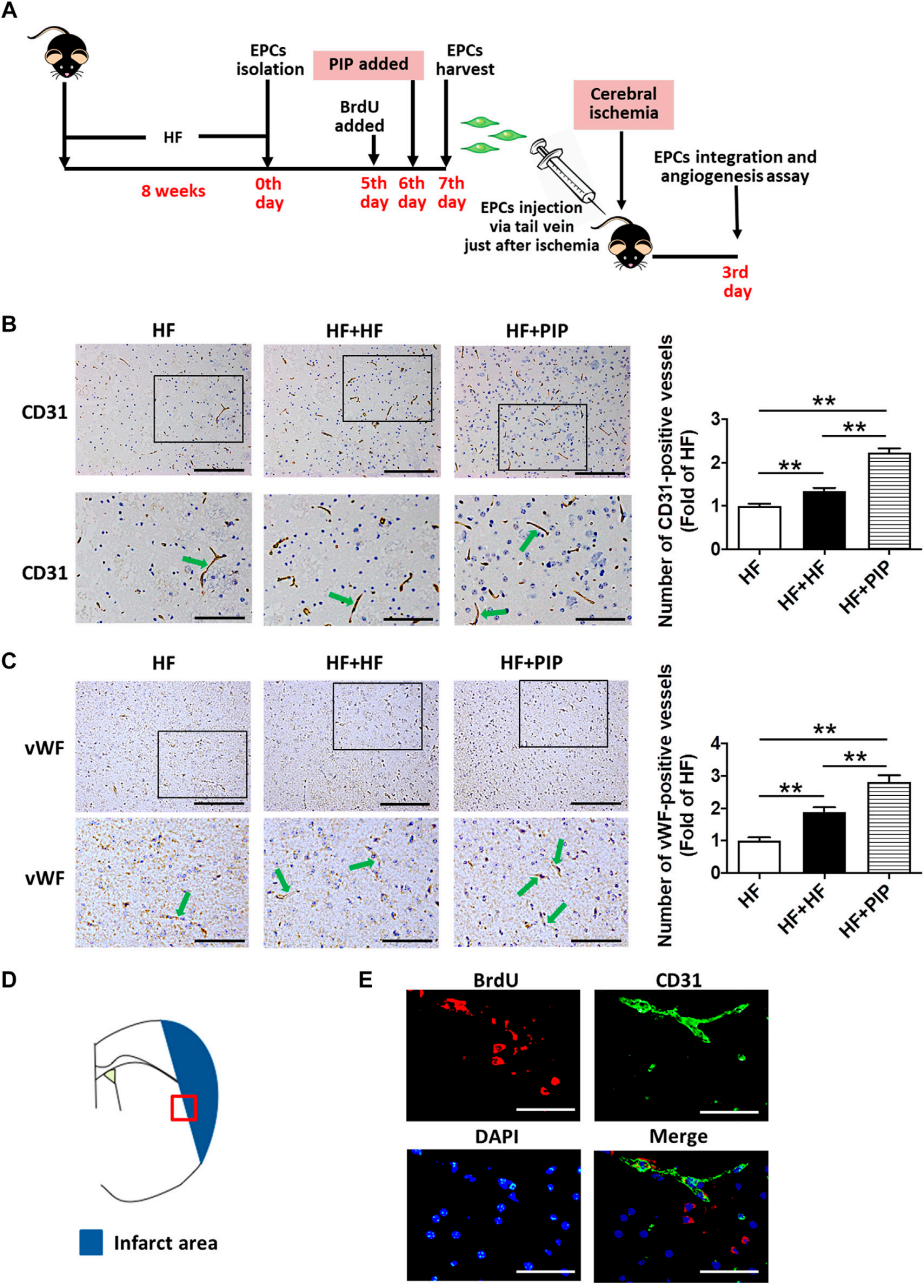
Incubation with piperlongumine increased the therapeutic effect of EPC from HFD-fed mice on angiogenesis promotion in HFD-fed mice. **(A)**, Surgical protocols: Male C57BL/6 mice (8 weeks) were fed with high-fat rodent chow for 8 weeks. Then, the EPCs were isolated from the HFD-fed mice. On day five of culture, BrdU were added to the media, and new medium supplemented with BrdU was replenished daily until day 7. Piperlongumine (1.0 μM) was added to EPCs on day 6 for 24 h. EPCs were harvested at the 7^th^ day and injected into the HFD-fed mice *via* tail vein just after the middle cerebral artery occlusion (1×10^6^ EPCs per mice). On day 3 after middle cerebral artery occlusion, the local angiogenesis in ischemic brain were determined. **(B)**, immunostaining for CD31 shows typical microvessels in the ischemic boundary area of ischemic brains. The bar graph shows increased capillary density in the two groups of EPCs-treated mice. Moreover, piperlongumine-incubated EPCs exerted a more positive effect on angiogenesis promotion compared to the EPCs without piperlongumine incubation (n = 9–12). Scale bars: 200 μm **(top)**; 100 μm **(bottom)**. **(C)**, immunostaining for vWF shows typical microvessels in the ischemic boundary area of ischemic brains. The bar graph shows increased capillary density in the two groups of EPCs-treated mice. Moreover, piperlongumine-incubated EPCs exerted a more positive effect on angiogenesis promotion compared to the EPCs without piperlongumine incubation (n = 8–9). Throughout, bars represent means; error bars represent SEM. Values were normalized to HF. **p < 0.01. Scale bars: 200 μm **(top)**; 100 μm **(bottom)**. **(D)**, The ischemic area and the ischemic boundary area of the ischemic brain, the red squares indicate the fields that were examined in this study. **(E)**, Photographs of ischemic boundary area indicate that the donor derived BrdU-positive EPCs (red fluorescence) were incorporated into CD31-positive microvessels (green fluorescence). Some BrdU-positive cells were found surrounding the microvessels. The nucleus was stained with DAPI (blue fluorescence). Scale bar: 50 μm. HF, HFD-fed mice; HF + HF, HFD-fed mice treated with EPCs; HF + PIP, HFD-fed mice treated with piperlongumine (1.0µM, 24 h)-incubated EPCs.

After 8 weeks of treatment, mice were used for EPCs isolation and assessment, or were subjected to permanent focal cerebral ischemia (Figures 2, 3, 4, 5, 6). Experiments were carried out in a random and blinded fashion. Experimental procedures were in accordance with the National Institutes of Health Guide for the Care and Use of Laboratory Animals (United States). All animal experiments complied with the ARRIVE guidelines.

### Isolation and Culture of Bone Marrow-Derived EPCs (BM-EPCs)

BM-EPCs were isolated and cultured according to the latest published methods, including ours (Wang et al., 2011; Dong et al., 2015). Briefly, mononuclear cells were obtained from mice femur and tibia as previously reported (Dong et al., 2017). Immediately, mononuclear cells were plated at a density of 5 × 10^6^ cells per well in EGM-2 (Lonza) in rat plasma vitronectin (Sigma, United States) coated six-well cell plates. Nonadherent cells were removed on the fourth day of culture, and remaining adherent cells were cultured for another 3 days. And then EPCs were either used for intracellular superoxide measurement, Intracellular nitric oxide measurement, function assays (Figures 2A, 4A) or for EPC transplantation (Figures 5, 6A).

### Cell Function Assays

*Migration assay.* EPCs were plated at a number of 5 × 10^4^ per well in the upper Boyden’s chamber with M199, while M199 supplemented with 50 ng/ml of VEGF and 10% FBS was placed in the lower chamber. After 24-h incubation time, migrated EPCs adhering to the lower membrane were fixed and then stained with 10 μg/ml of Hoechst 33258 (Sigma, United States). Then, the number of migrated EPCs were determined by counting the EPCs on the lower side of the membrane under a microscope at magnification ×100. In blinded analyses, for each sample, five images were taken from random fields (Dong et al., 2017; Peng et al., 2018).

*Tube formation assay.* A number of 5×10^4^ EPCs were plated per well of a 96-well cell culture plate precoated with growth factor-reduced Matrigel-Matrix (BD Biosciences, United States). After 6 h of incubation, images of tube morphology were taken by inverted microscope (Leica), and tube numbers were counted at five random fields at magnification of ×100 per sample (Dong et al., 2017; Peng et al., 2018).

*Adhesion assay.*1×10^4^ EPCs were plated per well of a 96-well plate precoated with 1 μg/ml vitronectin (Sigma, United States). After 4-h incubation time, nonadherent EPCs were removed, and residual adherent EPCs stained with 10 μg/ml of Hoechst (Hoechst 33258) and then fixed with 2% PFA. Adherent cells were counted at five random fields at magnification of ×100 per sample. Three wells were measured for each cell sample (Peng et al., 2018).

### Intracellular Superoxide (O_2_^−^) Measurement

O_2_^−^ levels were estimated using the fluorescent probe dihydroethidium (DHE) (Sigma, United States), a commonly used cell-permeable dye which is sensitive to O_2_^−^ and may be oxidized to the red fluorescent molecule ethidium. BM-EPCs were washed, collected and resuspended in M199 medium, and then labeled with 1 µM DHE for 40 min at 37°C in dark. After that, labeled EPCs were washed three times with 5%BSA/PBS and then fixed in 2% PFA. Finally, the labeled EPCs were analyzed by flow cytometry (Marrotte et al., 2010).

### Intracellular Nitric Oxide Measurement

NO levels were visualized using DAF-FM diacetate (4-amino-5-methylamine-2′, 7′-difluorofluorescein diacetate, Molecular Probes, United States). BM-EPCs were collected, washed and resuspended in PBS, and then labeled with 1 µM DAF-FM diacetate in dark. After incubation at 37°C for 40 min, labeled EPCs were washed three times with PBS and then fixed in 2% PFA. Finally, the labeled EPCs were analyzed by flow cytometry (Xie et al., 2010).

### Cerebral Ischemic Stroke Model

After 8 weeks of low-dose piperlongumine treatment, permanent focal cerebral ischemia was induced in mice according to previously described methods (Tamura et al., 1981; Welsh et al., 1987; Longa et al., 1989; Peng et al., 2018). Briefly, mice were anesthetized with 3.5% chloral hydrate at 0.1 ml/10 g body weight by intraperitoneal injection. A 0.5 cm skin incision was made between the left orbit and ear. Then, the left distal middle cerebral artery was exposed through a craniotomy and permanently cauterized above the rhinal fissure. The body temperature was maintained at 37 ± 0.5°C throughout the surgical procedure using a heating lamp and pad. Mice were treated with buprenorphine for analgesia on the day of surgery and twice daily thereafter for 72 h (Duricki et al., 2016; Calcagno et al., 2020). Behavioral assessment (including Body Asymmetry Test and Beam Test) was performed 3 days after middle cerebral artery occlusion, then mice were euthanized, and their brains were immediately removed. Infarct volume was determined by staining with 2,3,5-triphenyltetrazolium chloride (TTC) and was analyzed with ImageJ software. The experiments were performed in a blind and random fashion.

### Neurobehavioral Outcomes Analysis: Body Asymmetry Test and Beam Test

Neurological behavior analysis was carried out according to the protocols in previous studies and our previous work (Fan et al., 2010; Peng et al., 2018).

### Piperlongumine Incubation

To evaluate the direct effects of piperlongumine on EPCs dysfunction, BM-EPCs isolated from HFD-fed mice were incubated with piperlongumine (0.1 and 1.0 µM, 24 h), and then the migration function, adhesion function and tube formation function of cells were analyzed (Figure 4A). A dose of piperlongumine showing a better beneficial effect on EPC function will be adopted in the following tests about EPC incubation and EPC transplantation.

### Western Blot Analysis

Western blot analysis was carried out as previously described (Marrotte et al., 2010; Dong et al., 2017). In brief, collected EPCs culture media was concentrated using Amicon Ultra-4 centrifugal filters (Millipore) (3,000 rpm, 30 min, 4°C). Protein quantification was performed using the BCA assay (BCA Protein assay kit; Pierce Thermo Scientific), and equal amounts of total protein were fractionated by electrophoresis under denaturing conditions on 8% polyacrylamide gel and transferred to nitrocellulose membranes (Millipore). Proteins were detected by probing Western blots with antibodies specific to thrombospondin-1 (TSP-1) (Abcam) and thrombospondin-2 (TSP-2) (BD Transduction Laboratories™). Expression levels of the proteins were detected by Odyssey infrared image system (LI-COR), and the band intensities were quantified with the NIH ImageJ software.

### EPCs Transplantation

To further interpret our findings, we checked whether the EPC-mediated ischemic angiogenesis contributed to the protection of piperlongumine against cerebral ischemic injury in HFD-fed mice and assess whether the incubation with piperlongumine increased the therapeutic effect of EPCs from HFD-fed mice on cerebral ischemic injury reduction, 1×10^6^ BM-EPCs with or without piperlongumine incubation were systemically administrated to HFD-fed mice just after middle cerebral artery occlusion, and equivalent volume of vehicle (PBS) was administered to control mice (Figures 5, 6A) (Fan et al., 2010; Peng et al., 2018). At day 3 after stroke, behavioral tests were performed, and then the ischemic brains were serially cut and stained with 2% TTC for 5min (55°C) to determine the infarct area (Dong et al., 2015).

Additionally, at day 3 after middle cerebral artery occlusion (Figure 6A), the mice were anesthetized and the ischemic brains were flushed with PBS and perfusion in 4% PFA, before being dehydrated and embedded in paraffin. Serial sections 6 µm thick were cut in the coronal plane from paraffin-embedded blocks. Every 10th section was processed for immunohistochemical staining. Immunostaining was performed against CD31 antibody (BD Biosciences) or von Willebrand factor (vWF) to detect the angiogenesis in the area of ischemic boundary (Zhang et al., 2002; Eskilsson et al., 2016; Peng et al., 2018).

### *In Vivo* EPC Integration in HFD-Fed Mice

The homing of labeled EPCs into the ischemic boundary area of ischemic brain was analyzed using immunofluorescence study with 5-bromo-2′-deoxyuridine (BrdU)-labelled EPCs as published methods (Peng et al., 2018). On day five of culture, BrdU (1:100) were added to the media, and new medium supplemented with BrdU was replenished daily until day seven. Thereafter, the labeled EPCs were collected and resuspended in PBS and were then transplanted into mice (1 × 10^6^ cells/mouse) *via* the tail vein just after middle cerebral artery occlusion (Dong et al., 2017; Peng et al., 2018).

On day 3 after stroke, the mice were anesthetized, and the ischemic brains were exsanguinated by transcardial perfusion with PBS and perfusion fixed with 4% PFA at 4°C overnight. After being washed in fresh PBS, the ischemic brains were dehydrated, cleared and embedded in paraffin. Serial sections (6 µm thick) were cut in the coronal plane from the paraffin-embedded block. Every 10th coronal section was chosen for immunohistochemical staining (total three sections each) (Zhang et al., 2002; Chen et al., 2005; Dong et al., 2015). These sections were stained with anti-CD31 antibody at 4°C overnight, followed by BrdU antibody. Secondary antibodies applied were conjugates of Alexa Fluor 488 or Cy3. Finally, sections were counterstained with DAPI to visualize the nuclei (Dong et al., 2017; Peng et al., 2018).

### Statistical Analysis

Data are expressed as mean ± SEM. Statistical analysis was performed using one-way ANOVA followed by Tukey post hoc analysis. A value of *p* < 0.05 was considered statistically significant.

## Result

### Effects of Low-Dose Piperlongumine Administration on Body Weight, Fasting Blood Glucose and Lipid Levels in HFD-Fed Mice

There was no significant difference in food intake (Figure 1A) and body weight (Figure 1B) among all the groups. As shown in Figure 1C, fasting blood glucose levels did not differ among all the groups after 8 weeks of low-dose piperlongumine treatment. Compared to control mice, a significant increase in the serum levels of TC, TG, LDL-c and HDL-c were observed in the HFD-fed mice. However, 8 weeks treatment with low-dose piperlongumine showed no effect on the blood levels of TC, TG, LDL-c and HDL-c in HFD-fed mice (Figures 1D–G).

### Low-Dose Piperlongumine Administration Rescued EPC Dysfunctions in HFD-Fed Mice

After 8 weeks of low-dose piperlongumine treatment, the functions of BM-EPC were tested in HFD-fed mice (Figure 2A). Compared with control mice, BM-EPC functions were markly impaired in HFD-fed mice. However, an 8 weeks low-dose piperlongumine treatment markedly ameliorated the BM-EPC functions in HFD-fed mice (Figures 2B–D).

To investigate the potential mechanisms underlying low-dose piperlongumine administration protecting EPC functions, the level of intracellular O_2_^−^ and intracellular NO of EPCs were examined in HFD-fed mice after 8 weeks low-dose piperlongumine administration (Figure 2A). Compared to control, intracellular O_2_^−^ level of EPCs from HFD-fed mice was markedly increased. However, it was significantly attenuated in EPCs from low-dose piperlongumine treated HFD-fed mice when compared with that of the HFD-fed mice (Figure 2E). As shown in Figure 2F, intracellular NO level of EPCs from HFD-fed mice was markedly attenuated compared to that in control mice, which was rescued in EPCs from HFD-fed mice treated with low-dose piperlongumine.

### Low-Dose Piperlongumine Administration Protected Against Cerebral Ischemic Injury in HFD-Fed Mice

Then, we assessed whether the improvement of EPC functions produced by low-dose piperlongumine treatment would lead to a reduction of cerebral ischemic injury in HFD-fed mice. After 8 weeks of treatment, mice were subjected to middle cerebral artery occlusion (Figure 3A). We determined the cerebral ischemic injury and funtional outcomes 3 days after the onset of cerebral ischemia. It was found that high-fat diets increased infarct volume and neurological deficits in settings of stroke in mice. However, the cerebral infarct volume was markedly reduced, and the neurobehavioral outcomes were greatly improved in low-dose piperlongumine treatment HFD-fed mice compared with HFD-fed mice (Figures 3B–E). Taken together, low-dose piperlongumine treatment could protect against cerebral ischemic injury in HFD-fed mice.

### Incubation With Piperlongumine Rescued the Impaired Functions of EPCs From HFD-Fed Mice

To determine whether piperlongumine exerted direct beneficial effects on functions of EPCs from HFD-fed mice, we further performed cell function assays and TSP-1/2 level assessment in EPCs (Figure 4A). EPCs from HFD-fed mice were incubation with 0.1 and 1.0 μM piperlongumine for 24 h, and it was showed that the migration function of EPCs was dose-dependently improved after incubation with piperlongumine (Figure 4B). As 1.0 μM piperlongumine showed a better beneficial effect on EPC migration function, this dose of piperlongumine was adopted in the follow-up studies about EPC incubation and EPC transplantation. Compared with the EPCs from control mice, EPC functions were greatly impaired in HFD-fed mice, and incubation with piperlongumine (1.0 μM, 24 h) rescued the impaired functions of EPCs from HFD-fed mice (Figures 4C–E). In addition, the TSP-1 expression level in EPCs from HFD-fed mice was increased ∼2.7-fold compared with that of the control group, which was reduced by co-incubation with piperlongumine (1.0 μM, 24 h) (Figure 4F). The TSP-2 expression level in EPCs from HFD-fed mice was also enhanced compared with that of the control group, which was decreased by co-incubation with piperlongumine (1.0 μM, 24 h) (Figure 4F).

### Incubation With Piperlongumine Increased the Therapeutic Effect of EPC From HFD-Fed Mice on Cerebral Ischemic Injury Reduction and Angiogenesis Promotion in HFD-Fed Mice

To further determine whether the EPC-mediated ischemic angiogenesis contributed to the protection of piperlongumine against cerebral ischemic injury in HFD-fed mice and assess whether the piperlongumine increased the therapeutic effect of EPCs from HFD-fed mice on cerebral ischemic injury reduction, 1×10^6^ EPCs from HFD-fed mice with or without piperlongumine (1.0 μM, 24 h) incubation were systemically administrated to HFD-fed mice just after middle cerebral artery occlusion, and control HFD-fed mice received equal volume of vehicle (Figures 5, 6A). As shown in Figures 5B–E, EPCs or piperlongumine-incubated EPCs transplantation attenuated infarct volume in HFD-fed mice and the corresponding neurobehavioral outcomes were significantly improved in the two groups of EPC-treated mice compared with controls. However, piperlongumine-incubated EPCs showed a stronger therapeutic efficacy against cerebral ischemic injury than control EPCs from HFD-fed mice (*p* < 0.01).

Furthermore, the angiogenesis in ischemic brain was assessed on day 3 after middle cerebral artery occlusion. CD31 is mainly expressed in endothelial cells and is an endothelial marker. Endothelial cells specifically produce vWF, a marker that stains newly formed vessels (Yu et al., 2019). Using endothelial CD31 immunostaining as an index of capillary formation, we found that piperlongumine-incubated EPCs from HFD-fed mice exerted a markedly stronger effect on angiogenesis promotion compared to the EPCs from HFD-fed mice (*p* < 0.01) (Figure 6B). This was further verified and quantified by vWF immunohistochemistry. Likewise, a stronger effect of the piperlongumine-incubated EPCs from HFD-fed mice on angiogenesis promotion was detected as compared with the EPCs from HFD-fed mice (Figure 6C).

To further validate that BrdU-positive EPCs already successfully homing to the area of ischemic boundary, the sections were stained for CD31 and BrdU. As showed in Figures 6D,E, inspection of microvessels (labeled with CD31 immunostaining) proximal to the ischemic boundary area of ischemic brain in the mice with transplantation of EPCs revealed a conspicuous accumulation of BrdU-labeled cells (red). Previous studies have found that donor-derived BM-EPCs participate in angiogenesis in ischemic hindlimb and ischemic brain (Marrotte et al., 2010; Peng et al., 2018). Our finding demonstrated that the donor-derived EPCs could integrate into the area of ischemic boundary and participate in angiogenesis in HFD-fed mice.

These findings collectively demonstrated that the transplanted EPCs could incorporated into the area of ischemic boundary, promote local neovascularization capacity and protect against the cerebral ischemic injury in HFD-fed mice, and piperlongumine increased the therapeutic effect of EPCs from HFD-fed mice on angiogenesis promotion and cerebral ischemic injury reduction.

## Discussion

This study showed the first evidence that low-dose piperlongumine, which did not affect blood lipid levels, rescued EPC dysfunction and reduced cerebral ischemic injury in HFD-fed mice.

EPCs, as important precursors of endothelial cells, have been shown to participate in vascular formation, and have been used to successfully restore endothelial function and enhance neovascularization capacity in ischemic brain (Xie et al., 2010; Dong et al., 2017). However, studies indicate that dyslipidemia affected EPC survival and function (Botham and Wheeler-Jones, 2013; Cui et al., 2015). The present study demonstrated that low-dose piperlongumine could rescue impaired EPC functions in HFD-fed mice. Furthermore, we found that piperlongumine increased the therapeutic effect of EPCs from HFD-fed mice on angiogenesis promotion and cerebral ischemic injury reduction in HFD-fed mice, and the donor-derived EPCs homed to the recipient ischemic brain. Therefore, the restoration of impaired EPC functions may partly contribute to the protection of low-dose piperlongumine against cerebral ischemic injury in HFD-fed mice. However, the study about the protective effect of piperlongumine on neurological function is still limited in the present work, which is a major limitation and remains to be investigated in further studies.

It has been shown that the markedly elevated levels of intracellular O_2_^−^ and significantly reduced levels of intracellular NO may represent a major mechanism underlying EPC dysfunction (Xie et al., 2010), and the decreased NO production or increased O_2_^−^ production may upregulate TSP-1 and TSP-2 expression in cultured EPC (Xie et al., 2010; Bae et al., 2013; Xin et al., 2016). Furthermore, the upregulation of TSP-1 and/or TSP-2 could impair EPC function (Xie et al., 2010; Bae et al., 2013; Dong et al., 2017), inhibit angiogenesis and modulate endothelial cell migration, proliferation, survival and apoptosis (Lawler and Lawler, 2012). Thus, the increased NO levels and the decrease of O_2_^−^ levels together with the reduction of TSP-1 and TSP-2 levels, as observed in the present study, might partly contribute to the protective effect of piperlongumine on EPC function in HFD-fed mice. In addition, the anti-inflammatory properties of piperlongumine *via* the inhibition of MAPK and the NF-κB pathways have been demonstrated in human umbilical vein endothelial cells (Lee et al., 2013). Therefore, piperlongumine might also act through anti-inflammatory mechanism to rescue EPC dysfunction in HFD-fed mice, which need to be validated in future work.

Piperlongumine is a biologically active component from long pepper, which has been used in traditional medicine, including the Indian Ayurvedic system of medicine and the folk medicine of Latin America (Tripathi and Biswal, 2020). The infusion of long pepper root is used to induce expulsion of the placenta after birth. Other traditional uses of long pepper include treating tumors, diseases of the spleen, malaria, viral hepatitis, bronchitis, cough, asthma, respiratory infections, stomachache, and gonorrhea (Bezerra et al., 2013). Many *in vitro* and *in vivo* studies have reported pharmacological activities of piperlongumine include antidiabetic, antiplatelet aggregation, anti-atherosclerotic, antimetastatic, antinociceptive, antibacterial, antitumor (Park et al., 2008; Bezerra et al., 2013; Mgbeahuruike et al., 2019; Yoval-Sánchez et al., 2020). Our research, for the first time, found that low-dose piperlongumine could rescue impaired EPC function and reduce cerebral ischemic injury in HFD-fed mice. Given that 8 weeks treatment with low-dose piperlongumine showed no significant effect on the blood levels of TC, TG, LDL-c and HDL-c in HFD-fed mice, the protective effects of low-dose piperlongumine on cerebral ischemic injury in HFD-fed mice were not induced by decreasing blood lipid levels. Therefore, this study might provide a theoretical basis for the potential clinical application of piperlongumine for the treatment of cerebral ischemic injury in patients with hyperlipidemia.

Additionally, since EPCs has been demonstrated to play a critical role in regulating vascular repair and angiogenesis in various ischemic tissues besides ischemic brain, and EPC is well correlated with numerous cardiovascular risk factors (Fan et al., 2010; Marrotte et al., 2010; Zhao et al., 2013), the restoration of impaired EPC functions produced by low-dose piperlongumine treatment might also prevent other ischemic diseases except stroke in HFD-fed mice, such as acute myocardial infarction, peripheral vascular ischemic diseases, etc., which deserves to be explored in further studies.

**FIGURE 7:**
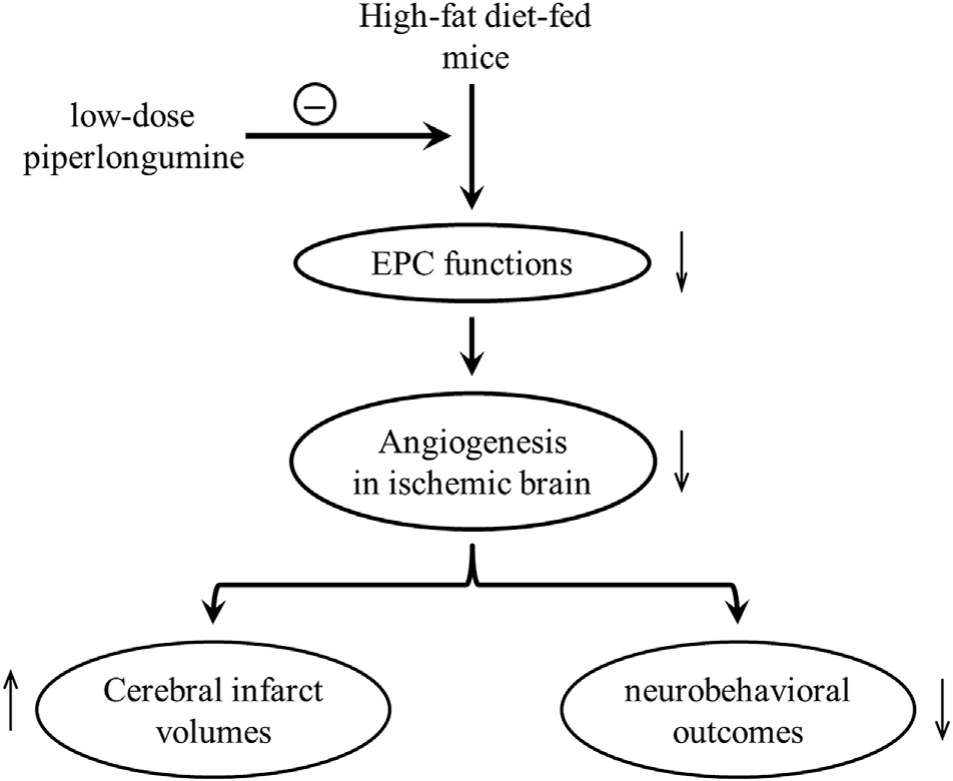
Putative mechanisms underlying low-dose piperlongumine reducing cerebral ischemic injury in HFD-fed mice. High-fat diet impaired endothelial progenitor cell function, decreased the ischemic angiogenesis, increased cerebral infarct volumes, and reduced the corresponding neurobehavioral outcomes in mice. Low-dose piperlongumine reduces cerebral ischemic injury *via* rescuing impaired EPC-mediated angiogenesis in HFD-fed mice.

In conclusion, low-dose piperlongumine could prevent cerebral ischemic injury in HFD-fed mice, which might be partly attributed to the improvement of EPC-mediated ischemic brain angiogenesis (Figure 7). This result implies that treatment with low-dose piperlongumine might be a potential option to prevent ischemic diseases (including stroke) in patients with hyperlipidemia, and priming with piperlongumine might be a feasible way to improve the efficacy of EPC-based therapy for ischemic diseases.

## Data Availability

The original contributions presented in the study are included in the article/Supplementary Material, further inquiries can be directed to the corresponding authors.
