# Supplementary material for: Low-Dose Piperlongumine Rescues Impaired Function of Endothelial Progenitor Cells and Reduces Cerebral Ischemic Injury in High-Fat Diet-Fed Mice
Source: Front Pharmacol. 2021 Nov 15;12:689880. doi: 10.3389/fphar.2021.689880 (PMC8634707; doi:10.3389/fphar.2021.689880)
Supplement: Supplementary file 1 [file DataSheet1.pdf]

Supplementary Table 1. Summary of statistical data

Figure	Test	Number of animals	Values	Statistical Test
1	Food intake	Con=8	Week 1: 22.4375;<br>Week 2: 23.2375;<br>Week 3: 21.42;<br>Week 4: 20.65;<br>Week 5: 21.34286;<br>Week 6: 21.94286;<br>Week 7: 20.38571;<br>Week 8: 20.87143	one-way ANOVA followed by Tukey post hoc analysis
		HF=11	Week 1: 23.94545;<br>Week 2: 24.02727;<br>Week 3: 25.61454;<br>Week 4: 22.68182;<br>Week 5: 25.7;<br>Week 6: 23.4125;<br>Week 7: 25.9;<br>Week 8: 22.81111	
		PIP=10	Week 1: 23.76;<br>Week 2: 24.5;<br>Week 3: 23.936;<br>Week 4: 25.08889;<br>Week 5: 24.33333;<br>Week 6: 25.21111;<br>Week 7: 23.94444;<br>Week 8: 21.64444	

	Body weight	Con=7-8	Week 0: 20.95±0.33;<br>Week 1: 22.63±0.40;<br>Week 2: 23.69±0.46;<br>Week 3: 24.70±0.65;<br>Week 4: 25.01±1.18;<br>Week 5: 25.76±0.62;<br>Week 6: 26.27±0.95;<br>Week 7: 26.86±1.30;<br>Week 8: 27.09±1.17	one-way ANOVA followed by Tukey post hoc analysis
		HF=9-11	Week 0: 22.16±0.28;<br>Week 1: 22.28±0.28;<br>Week 2: 25.75±0.33;<br>Week 3: 26.25±0.42;<br>Week 4: 26.15±0.45;<br>Week 5: 26.79±0.50;<br>Week 6: 27.73±0.54;<br>Week 7: 28.34±0.48;<br>Week 8: 28.37±0.41	
		PIP=9-11	Week 0: 21.88±0.32;<br>Week 1: 23.95±0.31;<br>Week 2: 24.76±0.47;<br>Week 3: 25.56±0.70;<br>Week 4: 26.21±0.54;<br>Week 5: 27.21±0.61;<br>Week 6: 28.26±0.62;<br>Week 7: 28.70±0.67;<br>Week 8: 28.28±0.57	
	Blood glucose	Con=7	Con=1.00±0.10	one-way ANOVA followed by Tukey post hoc analysis
		HF=9	HF=1.14±0.03	
		PIP=9	PIP=1.30±0.07	
	TC in blood	Con=5	Con=1.00±0.02	one-way ANOVA followed by Tukey post hoc analysis
		HF=9	HF=1.30±0.04	
		PIP=8	PIP=1.24±0.07	

	TG in blood	Con=5	Con=1.00±0.06	one-way ANOVA followed by Tukey post hoc analysis
		HF=9	HF=1.42±0.14	
		PIP=9	PIP=1.40±0.07	
	LDL-c in blood	Con=5	Con=1.00±0.02	one-way ANOVA followed by Tukey post hoc analysis
		HF=9	HF=1.47±0.13	
		PIP=9	PIP=1.33±0.06	
	HDL-c in blood	Con=5	Con=1.00±0.02	one-way ANOVA followed by Tukey post hoc analysis
		HF=9	HF=1.26±0.04	
		PIP=9	PIP=1.21±0.06	
2	Migrated Cells	Con=15	Con=1.00±0.07	one-way ANOVA followed by Tukey post hoc analysis
		HF=15	HF=0.42±0.02	
		PIP=16	PIP=0.94±0.05	
	Tube number	Con=20	Con=1.00±0.04	one-way ANOVA followed by Tukey post hoc analysis
		HF=20	HF=0.60±0.03	
		PIP=20	PIP=1.37±0.06	
	Adherent Cells	Con=25	Con=1.00±0.03	one-way ANOVA followed by Tukey post hoc analysis
		HF=20	HF=0.57±0.04	
		PIP=26	PIP=0.81±0.09	
	DHE fluoresence	Con=4	Con=1.00±0.01	one-way ANOVA followed by Tukey post hoc analysis
		HF=4	HF=1.24±0.02	
		PIP=5	PIP=0.83±0.02	
	DAF-FM fluoresence	Con=5	Con=1.00±0.05	one-way ANOVA followed by Tukey post hoc analysis
		HF=4	HF=0.41±0.07	
		PIP=4	PIP=1.08±0.18	
3	Infarct Volume	Con=8	Con=1.00±0.04	one-way ANOVA followed by Tukey post hoc analysis
		HF=8	HF=2.44±0.10	
		PIP=8	PIP=1.91±0.12	
	Asymmetic body swing rate	Con=8	Con=1.00±0.07	one-way ANOVA followed by Tukey post hoc analysis
		HF=8	HF=1.75±0.03	
		PIP=8	PIP=1.33±0.17	
	Time to cross beam	Con=8	Con=1.00±0.05	one-way ANOVA followed by Tukey post hoc analysis
		HF=8	HF=1.30±0.06	
		PIP=8	PIP=0.99±0.04	
4	Migrated Cells	HF=15	HF=1.00±0.07	one-way ANOVA followed by

		HF+0.1=15	HF+0.1=1.33±0.08	Tukey post hoc analysis
		HF+1.0=15	HF+1.0=1.76±0.13	
	Migrated Cells	Con=13	Con=1.00±0.07	one-way ANOVA followed by Tukey post hoc analysis
		HF=13	HF=0.62±0.04	
		HF+PIP=13	HF+PIP=0.94±0.05	
	Tube number	Con=15	Con=1.00±0.03	one-way ANOVA followed by Tukey post hoc analysis
		HF=20	HF=0.60±0.03	
		HF+PIP=15	HF+PIP=0.94±0.04	
	Adherent Cells	Con=17	Con=1.00±0.05	one-way ANOVA followed by Tukey post hoc analysis
		HF=20	HF=0.57±0.04	
		HF+PIP=17	HF+PIP=0.80±0.04	
	TSP-1 level	Con=7	Con=1.00±0.06	one-way ANOVA followed by Tukey post hoc analysis
		HF=7	HF=2.74±0.40	
		HF+PIP=7	HF+PIP=1.46±0.26	
	TSP-2 level	Con=6	Con=1.00±0.04	one-way ANOVA followed by Tukey post hoc analysis
		HF=6	HF=2.11±0.15	
		HF+PIP=6	HF+PIP=1.51±0.03	
5	Infarct Volume	HF=9	HF=1.00±0.02	one-way ANOVA followed by Tukey post hoc analysis
		HF+HF=9	HF+HF=0.84±0.04	
		HF+PIP=8	HF+PIP=0.66±0.03	
	Asymmetic body swing rate	HF=9	HF=1.00±0.02	one-way ANOVA followed by Tukey post hoc analysis
		HF+HF=9	HF+HF=0.73±0.06	
		HF+PIP=8	HF+PIP=0.60±0.04	
	Time to cross beam	HF=9	HF=1.00±0.05	one-way ANOVA followed by Tukey post hoc analysis
		HF+HF=9	HF+HF=0.81±0.01	
		HF+PIP=8	HF+PIP=0.66±0.01	
6	Capillaries (CD31)	HF=9	HF=1.00±0.05	one-way ANOVA followed by Tukey post hoc analysis
		HF+HF=12	HF+HF=1.34±0.08	
		HF+PIP=12	HF+PIP=2.23±0.09	
	Capillaries (vWF)	HF=9	HF=1.00±0.10	one-way ANOVA followed by Tukey post hoc analysis
		HF+HF=8	HF+HF=1.89±0.14	
		HF+PIP=8	HF+PIP=2.81±0.22	
